# Endocannabinoid LTD in Accumbal D1 Neurons Mediates Reward-Seeking Behavior

**DOI:** 10.1016/j.isci.2020.100951

**Published:** 2020-02-28

**Authors:** Ainhoa Bilbao, Daniela Neuhofer, Marja Sepers, Shou-peng Wei, Manuela Eisenhardt, Sarah Hertle, Olivier Lassalle, Almudena Ramos-Uriarte, Nagore Puente, Raissa Lerner, Pedro Grandes, Beat Lutz, Olivier J. Manzoni, Rainer Spanagel

**Affiliations:** 1 Behavioral Genetics Research Group, Heidelberg University, Medical Faculty Mannheim, 68159 Mannheim, Germany; 2 Institute of Psychopharmacology, Central Institute of Mental Health, Heidelberg University, Medical Faculty Mannheim, 68159 Mannheim, Germany; 3 INSERM U1249, Parc Scientifique de Luminy - BP 13 - 13273, Marseille Cedex 09, France; 4 Aix-Marseille University, Jardindu Pharo, 58 Boulevard Charles Livon, Marseille, 13007, France; 5 Cannalab, Cannabinoids Neuroscience Research International Associated Laboratory, INSERM-Indiana University, 107 S Indiana Avenue, Bloomington, IN 47405, USA; 6 Department of Neurosciences, Faculty of Medicine and Nursing, University of the Basque Country UPV/EHU, Barrio Sarriena s/n, 48940 Leioa, Spain; 7 Achucarro Basque Center for Neuroscience, Science Park of the UPV/EHU, Barrio Sarriena s/n, 48940 Leioa, Spain; 8 Institute of Physiological Chemistry, University Medical Center of the Johannes Gutenberg, University Mainz, Duesbergweg 6, 55099 Mainz, Germany

**Keywords:** Neuroscience, Behavioral Neuroscience, Molecular Neuroscience

## Abstract

The nucleus accumbens (NAc) plays a key role in drug-related behavior and natural reward learning. Synaptic plasticity in dopamine D1 and D2 receptor medium spiny neurons (MSNs) of the NAc and the endogenous cannabinoid (eCB) system have been implicated in reward seeking. However, the precise molecular and physiological basis of reward-seeking behavior remains unknown. We found that the specific deletion of metabotropic glutamate receptor 5 (mGluR5) in D1-expressing MSNs (D1^*miR*^mGluR5 mice) abolishes eCB-mediated long-term depression (LTD) and prevents the expression of drug (cocaine and ethanol), natural reward (saccharin), and brain-stimulation-seeking behavior. *In vivo* enhancement of 2-arachidonoylglycerol (2-AG) eCB signaling within the NAc core restores both eCB-LTD and reward-seeking behavior in D1^*miR*^mGluR5 mice. The data suggest a model where the eCB and glutamatergic systems of the NAc act in concert to mediate reward-seeking responses.

## Highlights

• mGluR5-D1-CB1-induced eCB-LTD mediates drugs of abuse and natural reward seeking
• eCB-LTD in D2-MSNs plays no important role in processing of reward-seeking responses
• Loss of eCB-LTD is a consequence of higher MAGL activity and lower CB1R expression
• Acute drug administration stops craving for alternative rewards on following days

## Graphical Abstract

**Figure:**
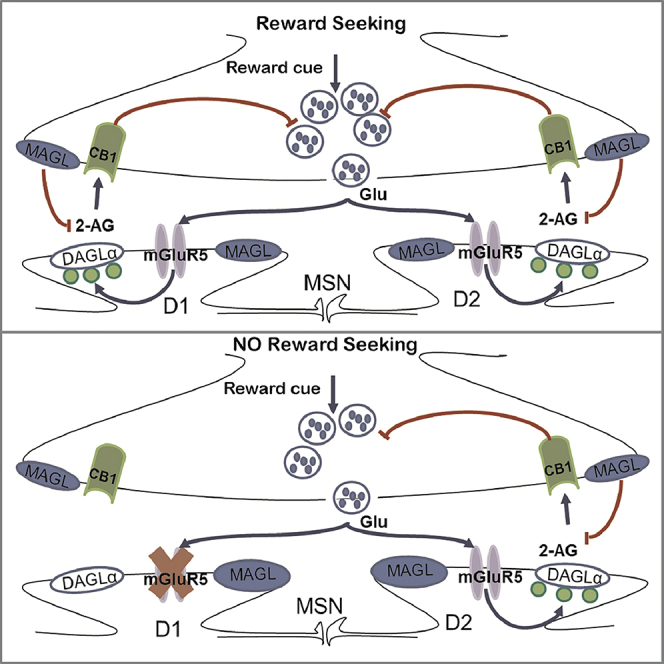

## Introduction

The brain reward system mediates motivational responses to natural rewards such as drinking, eating, and reproduction. Reinforcement learning for natural rewards depends on the formation of long-lasting conditioned associations that result in reward-seeking responses. This form of learning involves synaptic plasticity within the reward system, notably in medium spiny neurons (MSNs) of the nucleus accumbens (NAc) (Russo et al., 2010, Lüscher and Malenka, 2011). Drugs of abuse also act through the reward system, but the extent to which drug rewards and natural rewards share common neurobiological mechanisms within the reward system is barely studied.

One mechanism by which drugs of abuse alter synaptic plasticity in the reward system involves endocannabinoids (eCB). Stimulation of prelimbic cortex afferents at naturally occurring frequencies can cause long-term depression (LTD) of NAc glutamatergic synapses—an effect mediated by eCB release and presynaptic cannabinoid type 1receptor (CB1R) (Robbe et al., 2002, Zlebnik and Cheer, 2016, Araque et al., 2017). This form of eCB-mediated synaptic plasticity in the NAc depends on postsynaptic metabotropic glutamate receptor 5 (mGluR5) and is eliminated following exposure to drugs of abuse (Mato et al., 2004, Fourgeaud et al., 2004, Zlebnik and Cheer, 2016).

Although a direct involvement of this particular form of plasticity in reinforcement learning has so far not been demonstrated, there is increasing evidence that both mGluR5 and CB1R are critical for the expression of eCB-induced LTD and are involved in reward-seeking responses. Thus systemic pharmacological blockade of mGluR5 (Bäckström et al., 2004, Olive, 2009, Wang et al., 2013, Mihov and Hasler, 2016) or CB1R (De Vries et al., 2001, Cippitelli et al., 2005) reduces cue-induced reinstatement of drug-seeking responses. mGluR5 blockade also increases intracranial self-stimulation (ICSS) thresholds, suggesting a deficit in brain reward function (Cleva et al., 2012). The anatomical loci and neuronal mechanisms underlying these effects of CB1R and mGluR5 antagonists are still not well defined, but a specific role of CB1R in the NAc has been demonstrated for cocaine-, heroin-, and nicotine-seeking behavior in rodents (Xi et al., 2006, Kodas et al., 2007, Alvarez-Jaimes et al., 2008). Likewise mGluR5 in the NAc is also critically involved in drug-seeking behavior. Thus genetic deletion of mGluR5 in the NAc (Novak et al., 2010), intra-NAc administration of a selective mGluR5 antagonist (Knackstedt et al., 2014), and pharmacological inhibition of mGluR5 signaling via Homer proteins reduce cue-induced cocaine-seeking (Wang et al., 2013). Furthermore, combining sub-threshold doses of mGlu5R and CB1R antagonists also prevents alcohol-seeking behavior (Adams et al., 2010), suggesting an interaction between these two systems in mediating drug-seeking behavior. mGluR5 and CB1R blockade may also reduce seeking for food and other natural rewards. However, these findings are less consistent compared with those using drug rewards (Fattore et al., 2007, Sanchis-Segura et al., 2004, Olive, 2009, Sinclair et al., 2012, Watterson et al., 2013, Schmidt et al., 2015, Mihov and Hasler, 2016).

Two fundamental but unresolved issues are whether eCB-mediated synaptic plasticity that depends on presynaptic CB1R and postsynaptic mGluR5 in the NAc is causally involved in drug-seeking responses and whether the extent to which these synaptic changes also mediate natural reward-seeking responses; i.e., is there a common or distinct synaptic mechanism involved in drug and natural reward-seeking behavior. Here we mainly focused on e-CB-mediated synaptic plasticity in dopamine D1-receptor-containing MSNs, as several studies (Calipari et al., 2016, Hikida et al., 2010, Lobo and Nestler, 2011, Ma et al., 2018, Soares-Cunha et al., 2016) suggest that this neuronal population seems to be more likely involved in the formation of drug/natural reward-seeking behavior than D2-containing MSNs.

## Results

### mGluR5 in D1-Containing Neurons Mediate Drug- and Natural Reward-Seeking Behavior

**Figure 1:**
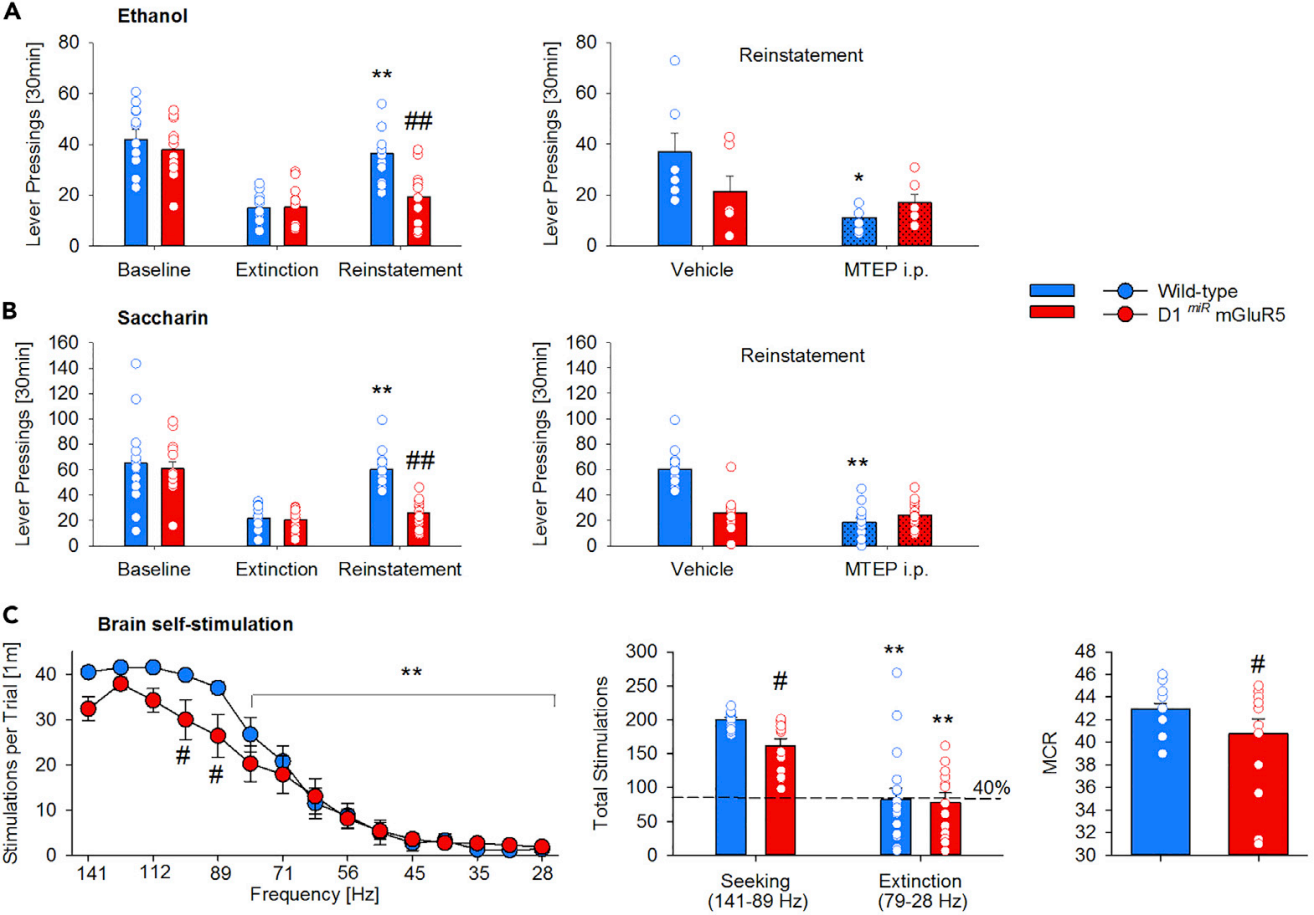
mGluR5 in D1-Containing Neurons Mediates Drug and Natural Reward-Seeking Behavior Selective genetic deletion of mGluR5 in D1 neurons results in a lack of cue-induced reinstatement of drug- (ethanol (A, left)) and natural (saccharin (B, left); brain stimulation (C)) reward-seeking behavior. Effect of systemic mGluR5 inhibition (MTEP, 20mg/kg, i.p.) on cue-induced reinstatement of ethanol- (A, right) and saccharin (B, right)-seeking behavior in wild-type (n = 6–14) and D1^*miR*^mGluR5 (n = 6–14) mice. The lower panel shows the brain stimulation data. During the testing phase of 15 descending frequencies, the first five frequencies were defined as the “seeking” component and the remaining 10 as the “extinction” component (left part). The seeking component of the rate frequency curve (C, middle) and the maximal reinforcement rate (maximum control rate (MCR), C, right) were significantly reduced in the D1^*miR*^mGluR5 (n = 13) mice compared with wild-type (n = 18) mice. All data represent mean from 15 mice +SEM. Two-way ANOVA; (∗) and (∗∗) corresponds to p < 0.05 and 0.0005, respectively vs. extinction, vehicle treatment (A and B) or the first five frequencies (141–89 Hz)/seeking (C); (#), (##) p < 0.05 and 0.0005 vs. wild-type mice, respectively.

Using a conditional mouse model with a knockdown of mGluR5 in dopamine D1-receptor-containing neurons (D1^*miR*^mGluR5 mice), we recently demonstrated that mGluR5 in this specific neuronal population mediates cue-induced reinstatement of cocaine-seeking behavior (Novak et al., 2010). Using this mouse model, we further asked if reward-seeking responses toward other drugs of abuse and natural rewards are also affected by this selective deletion of mGluR5 in D1-containing neurons. We first assessed ethanol-seeking responses in the reinstatement paradigm. D1^*miR*^mGluR5 mutants and wild-type littermates were trained under operant conditions. According to our standard protocol (Eisenhardt et al., 2015) mice were then trained for 15 days in 30-min sessions to lever press for ethanol with the presentation of contextual cues (S^+^/CS^+^) predictive of ethanol availability. All mice acquired stable lever pressing for ethanol (Figure S1A, two-way ANOVA, *genotype* effect: F_(1,22)_ = 0.3, p = 0.6; *genotype* × *time* interaction: F_(14,308)_ = 1.2, p = 0.3). Likewise, no genotype difference was observed during a 15-day extinction phase (Figure S1B, two-way ANOVA, *genotype* effect: F_(1,22)_ = 0.2, p = 0.7; *session* effect: F_(14,308)_ = 9.3, p < 0.0001). One day after the last extinction session, mice were tested for cue-induced reinstatement of ethanol seeking. The presentation of S^+^/CS^+^ significantly reinstated ethanol-seeking behavior in wild-type mice—an effect that was absent in mutant mice (Figure 1A left, two-way ANOVA, *genotype*: F_(1,22)_ = 6.1, p < 0.05, *cue* F_(2,44)_ = 41.6, p < 0.0001, and a *cue* × *genotype* interaction effect: F_(2,44)_ = 5.5, p < 0.01). These findings extend our previous observation of a similar phenotype with cocaine-associated cues (Novak et al., 2010) and demonstrate that mGluR5 in D1 neurons is a mediator of drug/cue memories and drug-seeking responses.

We next examined whether mGluR5 in D1 neurons is involved in the general processing of conditioned reward-seeking responses. For this purpose, a second group of mice was tested for its seeking behavior toward saccharin, a natural reward. During acquisition and extinction phases, again no genotype differences were observed for saccharin responding (Figure S1B and S1C two-way ANOVA, *genotype* effect: F_(1,27)_ = 0.8, p = 0.8 for acquisition and *genotype* effect: F_(1,27)_ = 2.2, p = 0.1 for extinction). Presentation of the S^+^/CS^+^ stimuli triggered reinstatement of saccharin-seeking behavior in wild-type mice but not in D1^*miR*^mGluR5 mice (Figure 1B left, two-way ANOVA, *genotype*: F_(1,27)_ = 10.7, p < 0.005, *cue* F_(2,54)_ = 40.6, p < 0.0001, and a *cue* × *genotype* interaction effect: F_(2,54)_ = 7.5, p < 0.005) demonstrating that mGluR5 in D1 neurons is also critical for natural reward-seeking responses.

Systemic administration of mGluR5 antagonists also reduces drug-seeking responses as well as saccharin-seeking responses (Bäckström et al., 2004, Olive, 2009, Watterson et al., 2013, Mihov and Hasler, 2016). If mGluR5 in D1-containing neurons would solely contribute to this systemic drug effect, reward-seeking responses in D1^*miR*^mGluR5 mice should not further be affected by mGluR5 antagonist. Therefore, we studied the effects of 2-methyl-6-(phenylethynyl)pyridine (MTEP 20 mg/kg) on cue-induced reinstatement of ethanol and saccharin seeking in our mutant mice. As expected, in comparison to vehicle, MTEP reduced the reinstatement response for ethanol-seeking behavior and saccharin-seeking behavior in wild-type mice. However, MTEP had no further effect in D1^*miR*^mGluR5 mice on the already reduced reinstatement (Figure 1A right, two-way ANOVA, *treatment* effect F_(1,10)_ = 6.7; p < 0.05 and Figure 1B right, *treatment* effect F_(1,27)_ = 48.6; p < 0.0001).

We further tested a third group of mutant mice for brain stimulation reward using the ICSS paradigm (Bilbao et al., 2015). Following ICSS training animals underwent the testing phase of 15 descending frequencies and all mice showed the expected frequency-dependent decrease in the number of stimulations per trial, which became significant from the sixth frequency on (Figure 1C, left, *frequency* effect F_(14,406)_ = 119.2; p < 0.0001). Because lower frequencies are less rewarding, and after having observed that from the sixth frequency on the performance of the mice started to decline, we defined the first five frequencies as the “seeking” component of the test and the remaining 10 as the “extinction” component. During the “seeking” component the performance of the D1^*miR*^mGluR5 mice was significantly lower than the response rate of the wild-type mice (*genotype* × *frequency* interaction effect F_(14,406)_ = 2.5; p < 0.01), whereas the “extinction” component was similar in all mice, performing 40% (similar to the extinction in operant conditions) of the baseline (Figure 1C, middle). Furthermore, compared with wild-type, D1^*miR*^mGluR5 mice also showed a significantly lower reinforcement rate (Figure 1C, right, t _(25)_ = 2.1; p < 0.05).

Finally, given the well-known crosstalk between mGluR5 in the MSN and presynaptic CB1R, we also studied the effects of the CB1R antagonist AM251 on cue-induced reinstatement of saccharin-seeking behavior in both wild-type and mutant mice. Similar to MTEP, systemic administration of AM251 reduced the reinstatement in wild-type mice, but not in D1^*miR*^mGluR5 mice (Figure S2, *treatment* effect F_(1,27)_ = 15.3; p < 0.001 and *treatment* × *genotype* interaction effect F_(1,27)_ = 27.6; p < 0.0001). In order to show that CB1R within the NAc is responsible for the observed effect in wild-type mice, a separate group of wild-type mice received an intra-accumbal administration of AM251. Again this brain-site-specific blockade of CB1R also reduced cue-induced reinstatement of saccharin seeking behavior (Figure S3, t_(8)_ = 3.7, p < 0.01).

In summary, mGluR5 in D1-containing neurons mediate in interaction with presynaptic CB1R cocaine- (Novak et al., 2010), ethanol-, saccharin-, and brain-stimulation-seeking responses. However, motor, emotional, and cognitive components can potentially affect reward-seeking responses (Sanchis-Segura and Spanagel, 2006). Therefore, in a series of control experiments, mice were tested for spontaneous locomotor activity, habituation to novelty, anxiety, short-term memory, and other D1-dependent responses. With the exception of a faster habituation to a novel environment (Figure S4B), all tested motor, emotional, or cognitive behaviors were normal in D1^*miR*^mGluR5 mice (Figure S4). Next we asked the question which synaptic mechanism could underlie the general lack of reward-seeking responses in D1^*miR*^mGluR5 mice.

### mGluR5 in D1 Neurons Mediates eCB-LTD

**Figure 2:**
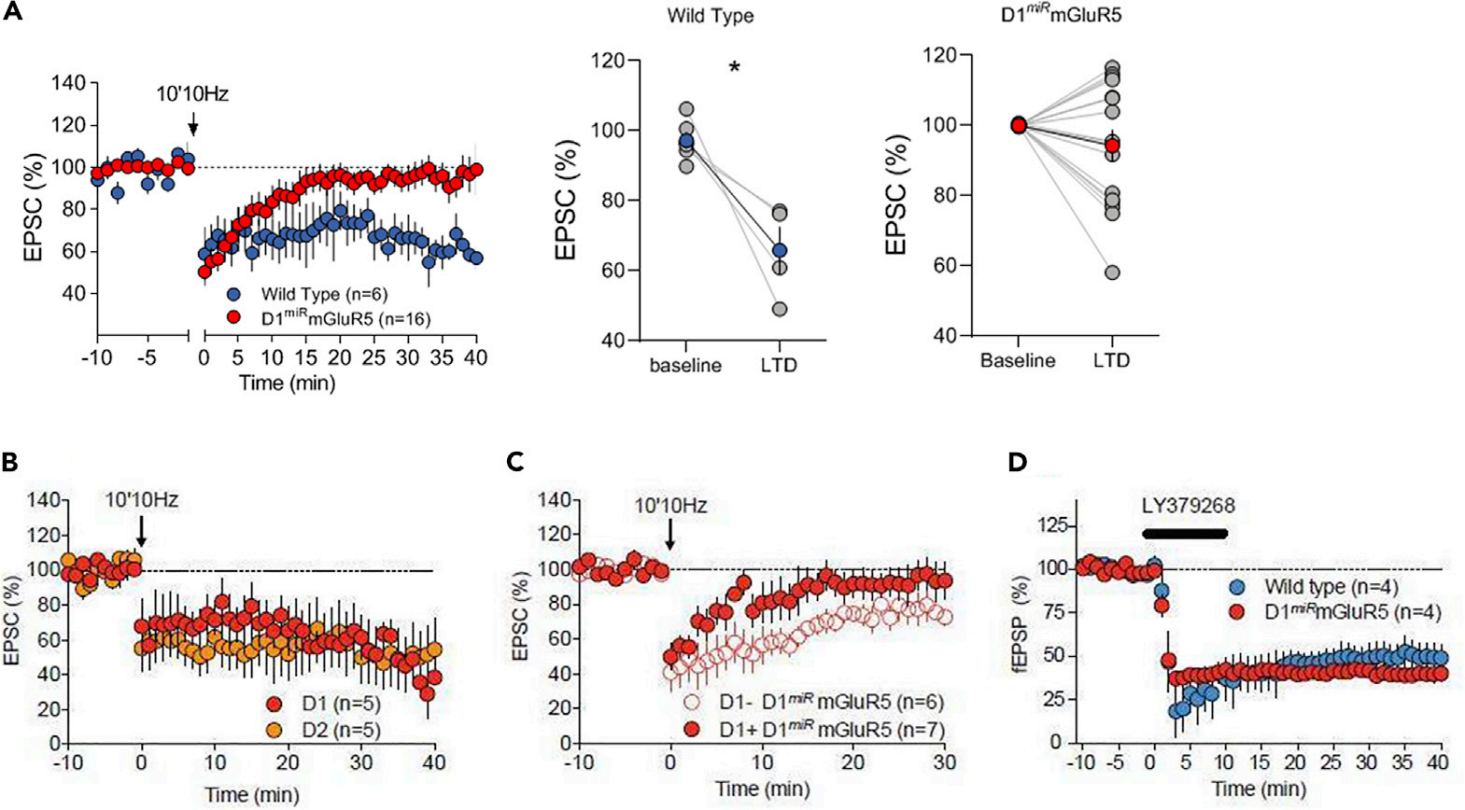
mGluR5 and CB1R in D1 Neurons Mediate eCB-LTD (A) Genetic downregulation of mGluR5 selectively in D1-MSNs abolishes eCB-mediated long-term depression in the NAc. Average time courses of mean EPSC (represented as percentage of the basal value) showing that in NAc slices prepared from wild-type (WT) and D1^*miR*^mGluR5 mice, low-frequency stimulation (10 min 10 Hz, indicated by arrow) induced LTD in WT (n = 6, blue circles) but not in D1^*miR*^mGluR5 mice (n = 16, red circles). Error bars indicate SEM, n = individual mouse. Adjacent to the timecourse, individual experiments (blue and red symbols) and group average (black symbols) before (baseline) and after LTD induction are shown. LTD was present in slices from WT mice (on the left) but in contrast, LTD was absent in D1^*miR*^mGluR5 mice (on the right). Error bars indicate SEM, n = individual mouse, ∗p < 0.05, Mann-Whitney U-test. (B) eCB-LTD is induced in MSNs from both the direct (D1 red) and indirect (D2 orange) pathways in wild-type mice. Retrogradely labeled direct and indirect pathway MSNs (see methods) were visualized by IR-DIC/epifluorescence microscopy and patch clamped. (C) In D1^*miR*^mGluR5 mice, eCB-LTD is selectively abolished in D1+ neurons, as 10-min Hz stimulation (arrow) induces LTD only in D1− (presumably D2+) NAc MSNs (empty red symbols) but is absent in D1+ neurons (filled red circles). (D) In contrast, bath application of the specific mGluR2/3 agonist, LY379268 (100nM), induces a profound LTD of fEPSP of similar amplitude in NAc of wild-type or D1^*miR*^mGluR5 mice. Average time courses of mean EPSC/fEPSP are represented as percentage of basal value.

We and others have shown that eCB-LTD requires the postsynaptic activation of mGluR5 to initiate retrograde eCB signaling (Robbe et al., 2002, Katona and Freund, 2012, Zlebnik and Cheer, 2016, Araque et al., 2017). Therefore, we examined basal synaptic transmission and eCB-mediated LTD in D1^*miR*^mGluR5 mice in NAc slices. Stimulation mimicking naturally occurring frequencies in NAc MSNs reliably induced a robust eCB-LTD in wild-type but not in D1^*miR*^mGluR5 mice (Figure 2A). Given that the deletion of mGluR5 is specific to D1-containing neurons (Novak et al., 2010), we suggest that the lack of eCB-LTD in D1^*miR*^mGluR5 mice contributes to the lack of reward seeking in these mice.

Previous reports showed also a prominent eCB-LTD in D2-MSNs in NAc (e.g. Shen et al., 2008, Kreitzer and Malenka, 2007, Grueter et al., 2010, Nazzaro et al., 2012), and this neuronal population may also contribute to reward-seeking responses (Soares-Cunha et al., 2016). We addressed this issue by specific labeling of MSNs that belong to either the indirect or direct dopamine pathways by using retrobeads. We patched retrogradely labeled direct (thereafter named D1-MSN) and indirect pathway (thereafter named D2-MSN) in wild-type mice and compared the capability of LTD induction in D1-or D2-MSNs in both wild-type and D1^*miR*^mGluR5 mice. In line with what has been previously shown eCB-LTD was expressed in direct and indirect pathway MSNs in wild-type animals (Figure 2B). Furthermore, in D1^*miR*^mGluR5 mice eCB-LTD was only present in D2-MSNs but not in D1-MSNs (Figure 2C). Taken together, these results suggest that eCB-LTD can be observed in both D1- and D2-MSNs and that we observe a selective lack of eCB-LTD in D1^*miR*^mGluR5 mice. If eCB-LTD in D2-MSNs would contribute to a decrease in reward-seeking responses, systemic or accumbal mGluR5 and presynaptic CB1R blockade should have led to an additional decline of responding in D1^*miR*^mGluR5 mice. This, however, was not the case (Figures 1, S2, and S3), which led us to the conclusion that eCB-LTD in D2-MSNs play no or an insignificant role in processing of reward-seeking responses, which is also in line with previous studies (Calipari et al., 2016, Hikida et al., 2010, Lobo and Nestler, 2011, Ma et al., 2018, Soares-Cunha et al., 2016).

We have previously shown that both CB1R and mGluR2/3 receptors share the same presynaptic machinery to induce LTD (Mato et al., 2005). To test if mGluR2/3-induced LTD is altered in D1^*miR*^mGluR5 mice we examined excitatory postsynaptic currents (EPSCs) in NAc MSNs following bath application of the mGluR2/3 agonist LY379268 and did not observe any genotype difference in LTD induction (Figure 2D), showing that the presynaptic LTD machinery is normal in D1^*miR*^mGluR5 mice.

**Figure 3:**
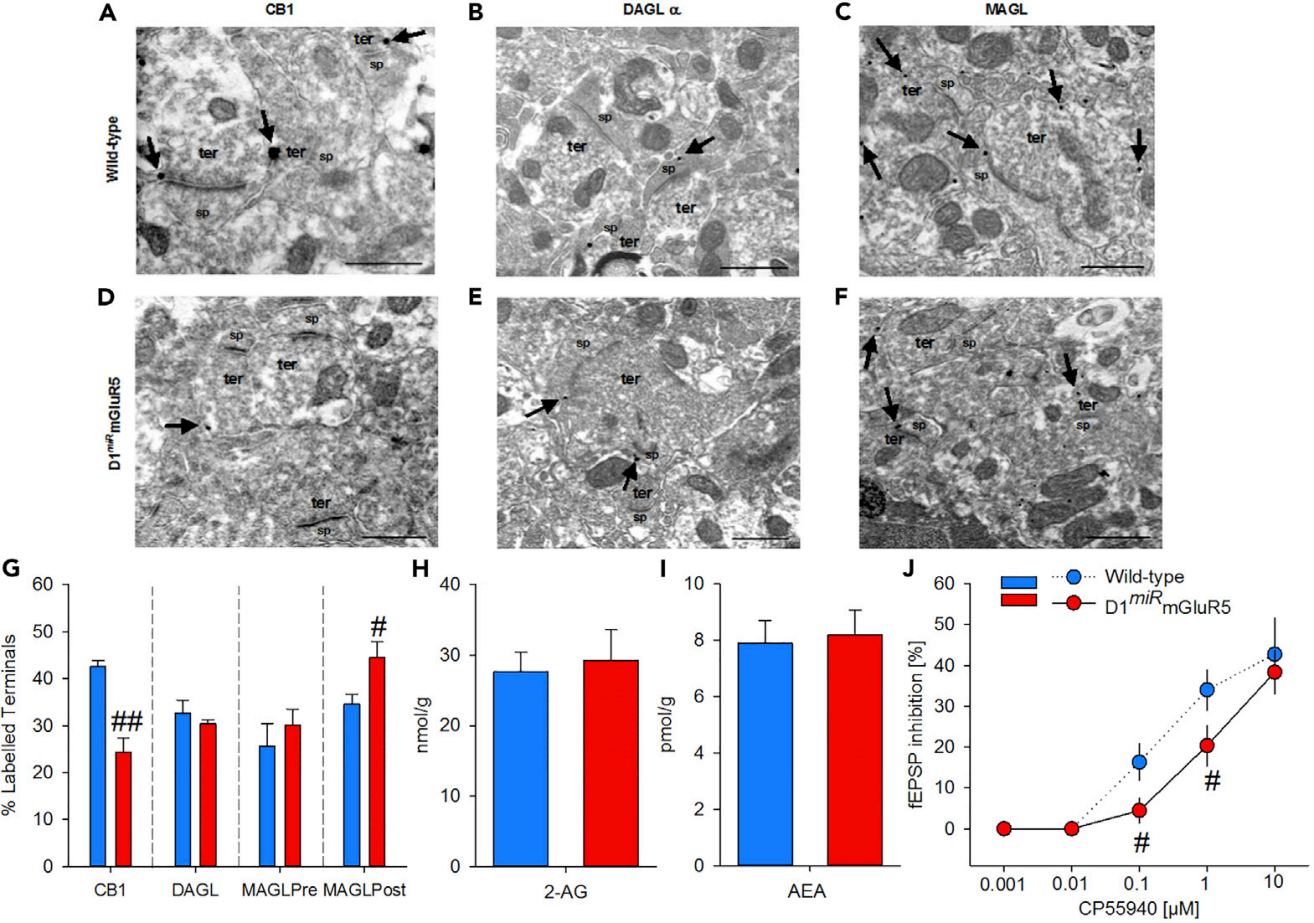
Ultrastructural Changes in the NAc in D1miRmGluR5 Mice (A–F) Ultrastructural immunolocalization of CB1R, DAGLα, and MAGL in the NAc of wild-type (n = 3, A–C) and D1^*miR*^mGluR5 (n = 3, D–F) mice assessed by preembedding silver-intensified immunogold method for electron microscopy. (A and D) CB1R immunoparticles (arrows) are distributed on perisynaptic and extrasynaptic membranes of axon terminals (ter) that make asymmetric synapses with dendritic spines (sp). (B and E) DAGLα immunolabeling is localized in dendritic spine membranes (arrows) away from the postsynaptic densities of asymmetric synapses. (C and F) MAGL shows a presynaptic and postsynaptic localization (arrows) on membranes of asymmetric presynaptic boutons and dendritic spines, respectively. (G) Proportion of presynaptic and postsynaptic profiles labeled by each antibody for wild-type and D1^*miR*^mGluR5 mutant mice. D1^*miR*^mGluR5 mice have fewer excitatory synaptic terminals with CB1R but more postsynaptic elements with MAGL receiving asymmetric synapses than wild-type mice. (H and I) Basal eCB concentrations of 2-arachidonoyl glycerol (2-AG, H) and anandamide (AEA, I) in the NAc are not affected in control (n = 10) and D1^*miR*^mGluR5 mice (n = 10). (J) Bath application of the cannabinoid agonist, CP55, 940, induces a dose-dependent inhibition of evoked fEPSPs recorded in the NAc synapses of wild-type mice, whereas in D1^*miR*^mGluR5, there is a shift to the right of the dose-response curve. Two-way ANOVA, (#) p < 0.05, (##) p < 0.0005 vs. wild-type. Scale bars 0.5 μm (A–G); picomoles or nanomoles/gram wet tissue +SEM (H and I).

Covariance of presynaptic CB1R functionality and eCB-LTD has been previously reported (Mato et al., 2004, Lafourcade et al., 2011). Thus, we thought to evaluate presynaptic CB1R efficiency in D1^*miR*^mGluR5 mice. The dose-response curve for the inhibition of evoked synaptic transmission in response to bath perfusion of the CB1R agonist CP55,940 was shifted to the right in NAc synapses of D1^*miR*^mGluR5 mice compared with wild-types (Figure 3J; CP55, 940 0.1 μM; p < 0.05) but the maximal inhibition was not significantly reduced (CP55, 940 10 μM; p = 0.54). Basal synaptic transmission was also not altered, as administration of a CB1R antagonist did not affect the basal synaptic transmission of both genotypes (Figure S5). These results suggest reduced CB1R expression in D1^*miR*^mGluR5 mice. We thus set out to study possible alteration in the eCB machinery on the ultrastructural level in our genetic mouse model.

### Deletion of eCB-LTD in the NAc Is a Consequence of Augmented MAGL Activity and Reduced CB1R Expression

CB1R and other components of the eCB system, such as the key enzymes monoacylglycerol lipase (MAGL) (Pan et al., 2011) and diacylglycerol lipase-α (DAGL-α) (Gao et al., 2010), as well as 2-arachidonylglycerol (2-AG) and other eCBs may have been influenced by the constitutive deletion of eCB-LTD in our genetic model. Here we used electron microscopy to analyze the distribution of CB1R, DAGL-α, and MAGL and mass spectrometry to quantify eCBs levels in the NAc of both wild-type and mutant mice.

As described previously (Puente et al., 2011), CB1R labeling was localized at presynaptic terminals making asymmetric synapses with dendritic spines and small dendrites (Figures 3A and 3D). A significant reduction of CB1R immunoparticles in D1^*miR*^mGluR5 mice compared with wild-type mice was found on the ultrastructural level within the NAc (Figure 3A, 3D, and 3G, t_(13)_ = 5.2, p <0.0005). Hence, reduced presynaptic expression of CB1R correlates with the reduced inhibition of CB1R-dependent excitatory transmission observed in the mutants (Figure 3J). The distribution of DAGL-α and presynaptic MAGL was in agreement with the subcellular pattern in other brain structures (Puente et al., 2011): DAGL-α immunolabeling was mostly distributed in spine head membranes postsynaptic to asymmetric synapses (Figures 3B and 3E), and MAGL immunoparticles were in membranes of excitatory synaptic terminals (Figures 3C and 3F). The proportion of immunoparticles for DAGL-α and presynaptic MAGL did not differ between genotypes (Figure 3G). MAGL was also found at dendritic spines, where it was significantly higher expressed in the mutants (Figure 3G, t_(14)_ = 2.5, p < 0.05). The significance of a differential distribution of this enzyme (pre- and postsynaptically) and the apparent paradox of increased postsynaptic expression with no changes in DAGL-α is not clear and might indicate non-overlapping functions of this enzyme, which could enable the establishment of two different pools of 2-AG, pre-stored and released, as it has been already suggested (Alger and Kim, 2011). Nevertheless, any possible functional relevance of increased postsynaptic MAGL activity in D1^*miR*^mGluR5 to eCB- mediated LTD is unlikely, as the hydrolysis by means of presynaptic MAGL has been proposed as a primary mechanism for 2-AG inactivation (Dinh et al., 2002).

The basal un-stimulated endogenous concentrations of 2-AG and anandamide (AEA) were normal in D1^*miR*^mGluR5 mice (Figures 3H and 3I: 2-AG: t_(18)_ = -0.3, p = 0.7; AEA: t_(18)_ = -0.2, p = 0.8). Neither 2-AG and AEA nor the metabolites 1-arachidonoyl glycerol (1-AG), arachidonic acid (AA), and other eCB congener concentrations, including oleoylethanolamide (OEA) and palmitoylethanolamide (PEA), (Figure S6) were affected. This finding is in line with the overall normal expression levels of the key enzymes in the mutant mice and supports the assertion that 2-AG levels are solely determined by the balance between production (DAGL- α) and presynaptic degradation by MAGL (Ohno-Shosaku et al., 2012). In summary, these ultrastructural data show that an augmented postsynaptic MAGL activity, combined with reduced CB1R and a lack of mGluR5, is the molecular alterations that result in a lack of eCB-LTD. One possibility to counteract these molecular alterations and to restore eCB-LTD is by increasing 2-AG levels (Bosch-Bouju et al., 2016).

### Enhancement of eCB Signaling Restores eCB-LTD and Reward-Seeking Responses in D1^miR^mGluR5

**Figure 4:**
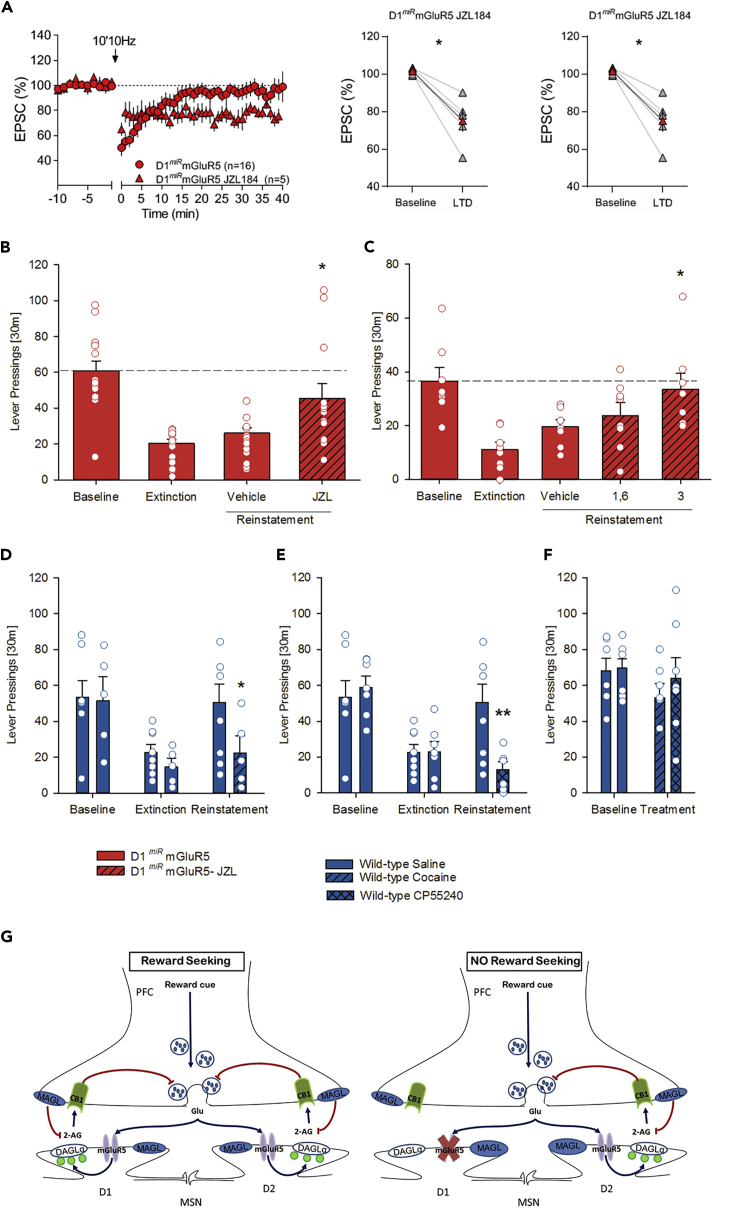
Rescue of Genetically Abolished eCB-LTD and Reward-seeking Behavior (A) Average time-courses of mean EPSC (represented as percentage of the basal value) showing that in NAc slices prepared from D1^*miR*^mGluR5 mice, low-frequency stimulation (10min 10 Hz, indicated by arrow) induced LTD in the presence of the MAGL inhibitor, JZL184 (1μM, red triangles, n = 5 mice) but not in untreated slices (red circles, n = 16 mice). Adjacent to the timecourse, individual experiments (red symbols) and group average (black symbols) before (baseline) and after LTD induction are shown. LTD is absent in untreated slices (on the left). In contrast, LTD was present in slices pretreated with JZL184 (on the right). Error bars indicate SEM, n = individual mouse, ∗p < 0.05, Mann-Whitney U-test. (B) Effects of MAGL inhibition on cue-induced reinstatement of saccharin-seeking behavior. Increasing 2-AG levels by administration of the MAGL inhibitor JZL 184 (16 mg/kg, i.p.; n = 15), produces a complete rescue of the reinstatement response in the D1*miR*mGluR5 mice. (C–F) (C) Intra-accumbal administration of JZL 184 into the NAc core of D1^*miR*^mGluR5 mice resulted in a dose-dependent rescue of the reinstatement response. Behaviorally, pharmacological blockade of eCB-LTD by cocaine (n = 6–7) or the CB1 agonist CP55,940 (n = 7) selectively abolished saccharin-seeking behavior triggered by the conditioned cues in wild-type mice (D, E), whereas it did not affect saccharin self-administration (F). (G) Accumbal D1 eCB-LTD is the physiological substrate for reward-seeking behavior. Graphic summary (G) showing the mechanism of reward seeking driven by accumbal D1-eCB-LTD. (Left) Presentation of the conditioned cue stimulates prefrontal cortico-accumbal glutamatergic neurons, glutamate is released into the synaptic space, and stimulation of mGluR5 of D1-containing MSNs triggers the synthesis and release of 2-AG through the DAGL pathway (blue arrows). Released 2-AG retrogradely activates CB1Rs in the presynaptic neuron (blue arrow), which in turn results in LTD, and thus a persistent reduction of synaptic neurotransmission (glutamate release, red arrow). This reduction on synaptic transmission might induce a “craving” state and participate to the seeking response. (Right) In D1^*miR*^mGluR5, presentation of the cue activates mGluR5-dependent perisynaptic signaling machinery (the synthesis and release of 2-AG through the diacylglycerol pathway) only in D2 neurons, not in D1 neurons. Without this D1-dependent LTD, seeking behavior is not triggered. Blue arrows indicate activation, and red arrows indicate inhibition. All data represent mean ± SEM. Two-way ANOVA, (∗) p < 0.05 and (∗∗) p < 0.01 vs. vehicle treatment, respectively.

The MAGL inhibitor JZL184 produces a selective blockade of 2-AG hydrolysis and thereby leads to an increase in 2-AG levels (Long et al., 2009, Bosch-Bouju et al., 2016). JZL184 (1 mM) restored eCB-LTD in the NAc in D1^*miR*^mGluR5 (Figure 4A). Likewise, systemic application of JZL184 prior to the reinstatement led to a saccharin-seeking response in D1^*miR*^mGluR5 mice (Figure 4B, t _(2.8)_ = −2.8, p < 0.05), which was comparable to the one observed in wild-type mice. In wild-type mice the reinstatement response was attenuated by JZL184 (Figure S7, t _(13)_ = 9.2, p < 0.0001), possibly due to the functional desensitization of CB1R in response to elevated 2-AG levels (Long et al., 2009) or due to the rewarding properties of enhanced endogenous 2-AG levels (Justinova et al., 2005), and subsequent CB1R stimulation. As a proof of concept experiments, we sought to demonstrate a direct role of the NAc by infusing JZL184 into this brain site. JZL184 dose-dependently restored the reinstatement response (Figure 4C, t _(7)_ = 3.4, p < 0.05 and t _(7)_ = 4.4, p < 0.01 for 1.6 and 3μg/0.5μL, respectively). Taken together, these data show that increasing 2-AG levels and thereby CB1R signaling in the NAc restores e-CB-LTD in D1^*miR*^mGluR5 mice and subsequently reward-seeking responses.

If eCB-LTD in D1-containing neurons is the physiological substrate of reward-seeking behavior, one should be able to manipulate reward-seeking by prior drug administration. Thus, it has been shown that already a single *in vivo* exposure to cocaine (Fourgeaud et al., 2004) or Δ9-THC (Mato et al., 2004) abolishes eCB-LTD in the NAc. Therefore, acute drug treatment that ablates eCB-LTD should impede reward seeking in wild-type mice. Indeed, a single cocaine or CP55,940 injection 24 h before reinstatement testing—a protocol that abolishes eCB-LTD—selectively inhibited cue-induced saccharin-seeking responses (Figure 4D, ANOVA, for cocaine: *reinstatement* effect: F_(2,22)_ = 11.9, p < 0.001; *reinstatement × treatment* interaction effect: F_(4,34)_ = 4.5, p < 0.01; Figure 4E, for CP55,940: *reinstatement* effect: F_(2,26)_ = 16.9, p < 0.0001; *reinstatement × treatment* interaction effect: F_(2,26)_ = 7.7, p < 0.005) but not self-administration (Figure 4F, *self-administration* effect: F_(1,11)_ = 3.7, p = 0.08; *treatment* effect: F_(1,11)_ = 0.3, p = 0.6) in wild-type mice. This is a finding with clinical implications, as it would suggest that acute forced drug administration abolishes craving for an alternative reinforcer on subsequent days. This observation deserves closer investigations, especially in relevance for human drug-taking behavior.

## Discussion

Here we propose a model (Figure 4G) in which the presentation of drug-conditioned cues activate excitatory afferents to the NAc and facilitate drug and natural reward-seeking responses by encoding reward-associated cues (Britt et al., 2012). This excitation leads to an increase of accumbal glutamate transmission (Kalivas, 2009) and activation of postsynaptic mGluR5 on D1-MSNs. Subsequent mGluR5-mediated eCB-LTD within the NAc leads then to an inhibition of cue-induced glutamate release and finally to a state of seeking for the reward-associated cue. The selective inhibition of glutamate transmission might not take place when key players such as CB1R and mGluR5 are pharmacologically blocked or genetically manipulated as in our D1^*miR*^mGluR5 mutants. Hence in D1^*miR*^mGluR5 mice, presentation of a reward-associated cue does not activate the mGluR5-dependent perisynaptic signaling machinery; i.e., the synthesis and release of 2-AG through the diacylglycerol pathway (Hashimotodani et al., 2007). A downregulation of CB1R from glutamatergic terminals ultimately results in an impairment of long-term control of glutamate release by a lack of D1-MSN mediated LTD induction. Without this LTD, seeking behavior in these mutant mice is not triggered.

Our data are compatible with the idea that both D1- and D2 (as well as D1/D2)-expressing neurons produce 2-AG in the NAc. We propose that in wild-type mice, D1 and D1/D2 MSNs are a principal source of the 2-AG that mediates eCB-LTD and reward-seeking responses. In our D1^*miR*^mGluR5 mutants, the production of 2-AG is insufficient to reach the threshold of LTD induction. In the presence of JZL184, 2-AG is augmented and sufficient to trigger LTD. This idea in line with a recent study shows that mGluR5 antagonism inhibits cocaine reinforcement and relapse by elevation of extracellular glutamate in the NAc via a CB1 receptor mechanism (Li et al., 2018).

The possibility that D2-MSN in the NAc core could have also contributed to the alterations in reward-seeking responses in mutant mice is unlikely because a previous study by Anderson et al. (2006) showed that cooperative activation of D1-like and D2-like dopamine receptors is required for the reinstatement of a drug-seeking behavior in the NAc shell, but not core. Furthermore, neither systemic nor accumbal mGluR5 or presynaptic CB1R blockade led to a further decrease in D1^*miR*^mGluR5 mutants (Figure 1, S2, and S3). This suggests that eCB-LTD in D2-MSNs plays no or only a minor role in the processing of reward-seeking responses, which is also in line with previous studies (Calipari et al., 2016, Hikida et al., 2010, Lobo and Nestler, 2011, Ma et al., 2018, Soares-Cunha et al., 2016).

In conclusion, we show that mGluR5 interacts with the eCB system to induce synaptic changes in D1 neurons in the NAc and that the resulting eCB-LTD mediates cue-induced reward-seeking responses. We propose that such molecular and synaptic events contribute to the common neural circuit adaptations that underlie the persistence of natural and drug reward memories. Cues paired with drugs of abuse can drive behavioral and physiological responses responsible for craving and relapse even after long periods of abstinence (Shaham et al., 2003, Sanchis-Segura and Spanagel, 2006), and similarly, cues conditioned to non-drug rewards such as highly palatable liquids and food, sex, and money induce similar network activity and contribute to overeating, obesity, gambling, and other forms of addictive behavior (Noori et al., 2016). Proposing that natural and drug rewards share the same molecular and physiological correlate for cue-induced reward-seeking responses, medications targeting mGluR5-dependent eCB-LTD, such as compounds that modulate endogenous 2-AG levels, may be useful in treating a variety of addictive behaviors.

### Limitations of the Study

The present study has two limitations. First, with our mouse model we could not well distinguish about the contribution of D1- and D2-MSNs in the processing of reward-seeking responses. Although our data strongly support the conclusion that eCB-LTD in D2-MSNs plays no important role in processing of reward-seeking responses, the use of D2miRmGluR5 mutants would have provided full evidence for this conclusion. Clearly, the generation of such a selective transgenic mouse model and its full behavioral, molecular, neuroanatomical, and physiological characterization as presented here for D1miRmGluR5 mutants would have been a major contribution on its own. Secondly, we did not perform slice experiments to test if saccharin exposure can abolish eCB-LTD. This research question was indeed of interest in regard to our previous publications where we showed that eCB-mediated synaptic plasticity in the NAc is eliminated following exposure to drugs of abuse (Mato et al., 2004, Fourgeaud et al., 2004, Zlebnik and Cheer, 2016). However, in the context of this study, this additional information would not have impact on our conclusions: the present work tested the hypothesis that eCB-LTD in D1 MSN mediates reward-seeking behavior and not that natural rewards would abolish this form of plasticity.

## Methods

All methods can be found in the accompanying Transparent Methods supplemental file.

## Supplemental Information

Document S1. Transparent Methods and Figures S1–S7
