# Supplementary material for: Endocannabinoid LTD in Accumbal D1 Neurons Mediates Reward-Seeking Behavior
Source: iScience. 2020 Feb 28;23(3):100951. doi: 10.1016/j.isci.2020.100951 (PMC7068121; doi:10.1016/j.isci.2020.100951)
Supplement: Document S1. Transparent Methods and Figures S1–S7 [file mmc1.pdf]

iScience, Volume *23*

Supplemental Information

Endocannabinoid LTD in Accumbal D1 Neurons Mediates Reward-Seeking Behavior

Ainhoa Bilbao, Daniela Neuhofer, Marja Sepers, Shou-peng Wei, Manuela Eisenhardt, Sarah Hertle, Olivier Lassalle, Almudena Ramos-Uriarte, Nagore Puente, Raissa Lerner, Pedro Grandes, Beat Lutz, Olivier J. Manzoni, and Rainer Spanagel

Supplemental Figure 1

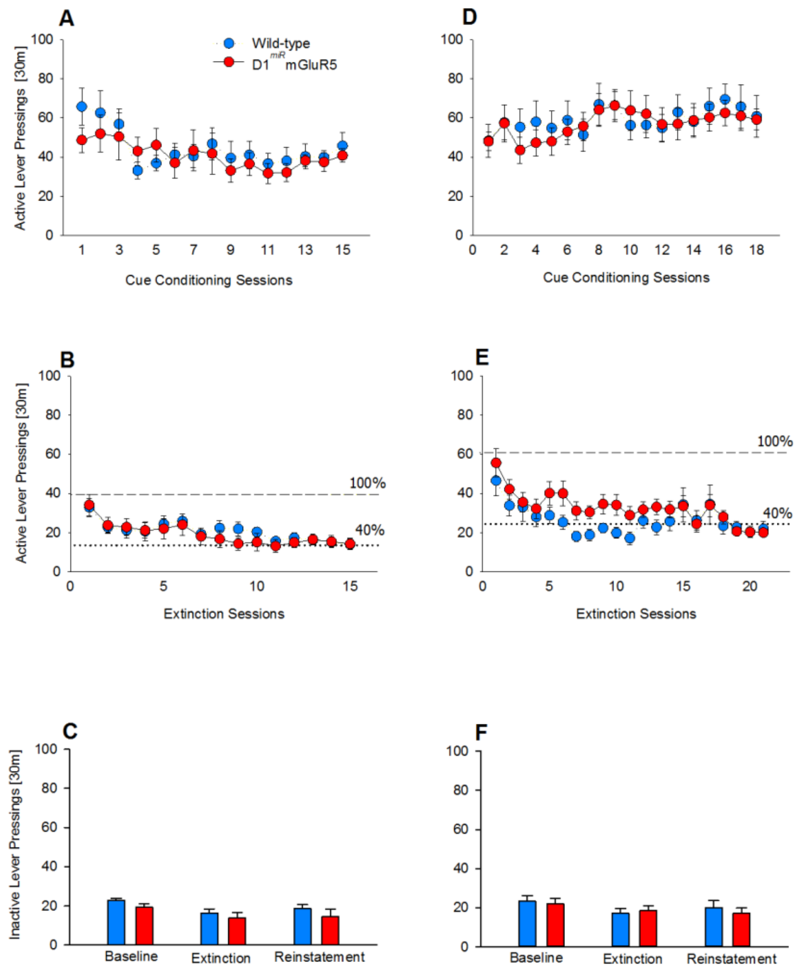

Figure S1. Active and inactive lever pressing for operant self-administration during training and extinction phases, related to Figure 1.
(A) Operant lever press responses across 15 daily sessions of contextual cues (S^+/CS^+) pairings with a 10% ethanol solution are stable and do not significantly differ between wild-type and D1^{miR}mGluR5 mice (n=12 per genotype). (B) Extinction is achieved after 15 sessions and does not differ between the two genotypes. (C) Inactive lever pressing during the S^+/CS^+ conditions (during the last 3 days of the training phase), the extinction phase and during the reinstatement test are similar in both genotypes (D) Operant lever responses for the natural reward saccharin did not differ in wild-type (n=14) and D1^{miR}mGluR5 mice (n=15). (E) Extinction is achieved after 21 sessions and does not differ between the two genotypes. (F) Inactive lever pressing during the S^+/CS^+ conditions (during the last 3 days of the training phase), the extinction phase and during the reinstatement test are similar in both genotypes. Data represent mean $\pm$ SEM.

Supplemental Figure 2

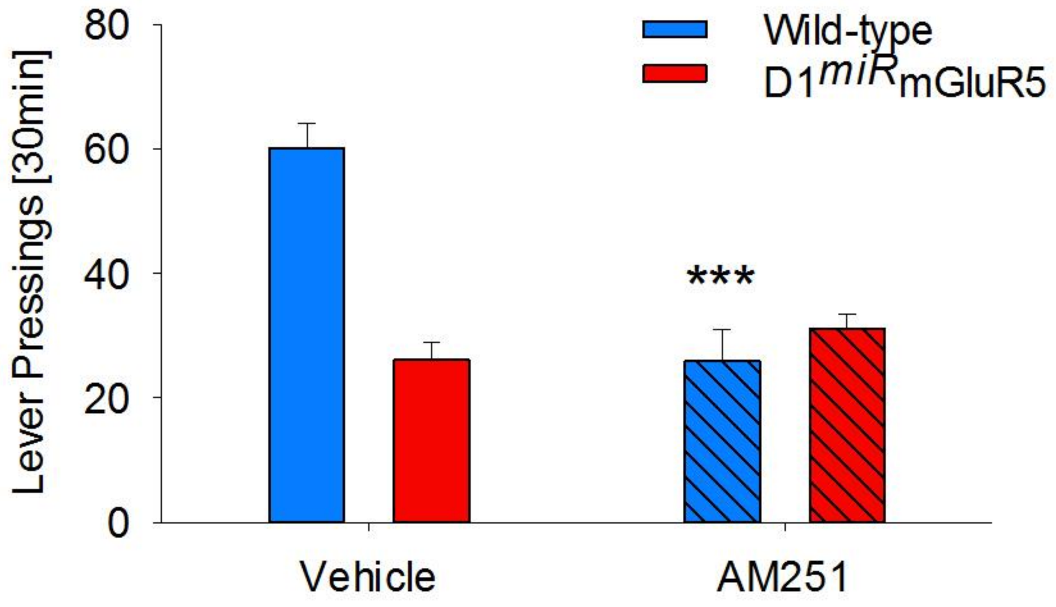

Figure S2. Effect of systemic administration of the CB1 antagonist AM251 on cue-induced reinstatement of saccharin-seeking behavior, related to Figure 1.

Systemic administration of AM251 reduced the reinstatement in wild-type mice, but not in D1^{miR}mGluR5 mice. Data represent mean ± SEM. Two-way ANOVA, (*) *P* <0.001 vs vehicle treatment.

Supplemental Figure 3

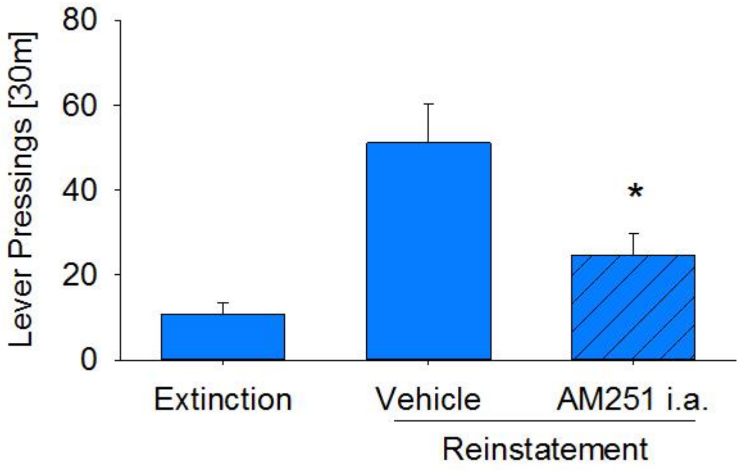

Figure S3. Effect of accumbal inhibition of CB1 on cue-induced reinstatement of saccharin seeking, related to Figure 1. Effect of accumbal inhibition of CB1 (1µg/0.5µl AM251, intra-accumbal) on cue-induced reinstatement of saccharin seeking in wild-type (n=9) mice. Data represent mean $\pm$ SEM. Two-way ANOVA, (*) P <0.001 vs vehicle treatment.

Supplemental Figure 4

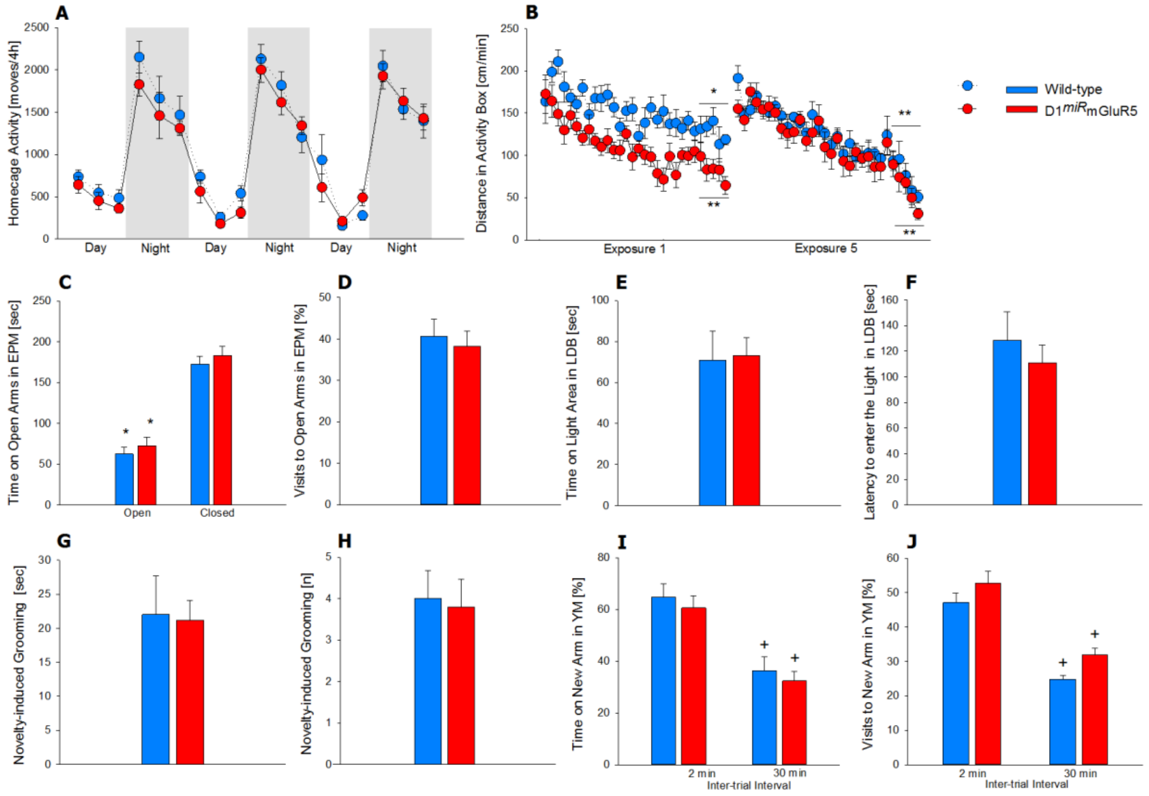

Figure S4. Locomotor activity, anxiety and short-term memory are intact in D1^{miR}mGluR5 mice, related to Figure 1.

(A) Spontaneous home cage locomotor activity measured by the e-motion system is indistinguishable between wild-type (n=7) and D1miRmGluR5 mice (n=9) (Two-way ANOVA, *genotype* effect ($F_{(1,84)}$=2.8, *P* <0.1). Both genotypes display typical diurnal rhythmicity with higher activity levels during the night phase compared with the resting, light phase of the day. Two-way ANOVA indicates a *phase* effect ($F_{(1,84)}$=430.5, p<0.0001), and all day points are significantly different from all night points (Newman-Keuls post-hoc test, *P*<0.05, not indicated). (B) D1^{miR}mGluR5 mice show a faster habituation to novelty in the activity boxes. During the first 30 min exposure (Exposure 1), both genotypes display decreased locomotor activation in the last 5 min compared to the first 5 min, indicating habituation to novelty (Two-way ANOVA, *time* effect: $F_{(9,126)}$=13, *P* <0.0001). However, this effect is more pronounced in D1^{miR}mGluR5 mice (*genotype* effect: $F_{(1,14)}$=10.4, *P* <0.01, Newman-Keuls post-hoc test indicates *P* <0.005 in wild-types and *P* <0.0001 in D1^{miR}mGluR5 mice comparing the first and the last 5 min of the exposure). After repeated exposures (Exposure 5) habituation processes are not different anymore between genotypes (Two-way ANOVA, *genotype* effect: $F_{(1,14)}$=1.6, *P* =0.2; *time* effect: $F_{(9,126)}$=37.3, *P* <0.0001; Newman-Keuls post-hoc test *P* <0.0001 for both genotypes comparing the first and the last 5 min of the exposure). (C-H) Anxiety-related behavior is not different between both genotypes. (C, D) Elevated plus-maze test. The time spent (C) and number of visits to the open arms (D) of the maze is almost identical in both genotypes ($t_{(51)}$=0.5, *P*=0.6). (E,F) similarly, in light-dark box, time spent in the light area (E) or the latency to enter the light

zone (F) is not different between wild-type and D1^{miR}mGluR5 mice ($t_{(53)} = -0.1$; P=0.9 and $t_{(53)} = 0.7$; P =0.5, respectively). (G, H) Novelty-induced grooming duration (G) and frequency (H) is indistinguishable between wild-type and D1miRmGluR5 mice ($t_{(15)} = 0.1$; P =0.9 and $t_{(15)} = 0.2$; P =0.8, respectively). (I, J) Evaluation of the short-term memory in the Y-maze test shows an intact performance in D1^{miR}mGluR5 mice, as indicated by the decreased time (I) and number of visits (J) displayed to the new arm 30 min after the first exposure (inter-trial-interval 30) (Two-way ANOVA, *inter-trial interval* effect: $F_{(1,30)} = 56$; P <0.0001 and $F_{(1,30)} = 82.6$; P <0.0001 for I&J, respectively). Data represent mean $\pm$ SEM. Two-way ANOVA, * P <0.005 and ** P <0.0001 compared with first 5 minutes; + P <0.005 compared with 2 min inter-trial interval.

Supplemental Figure 5

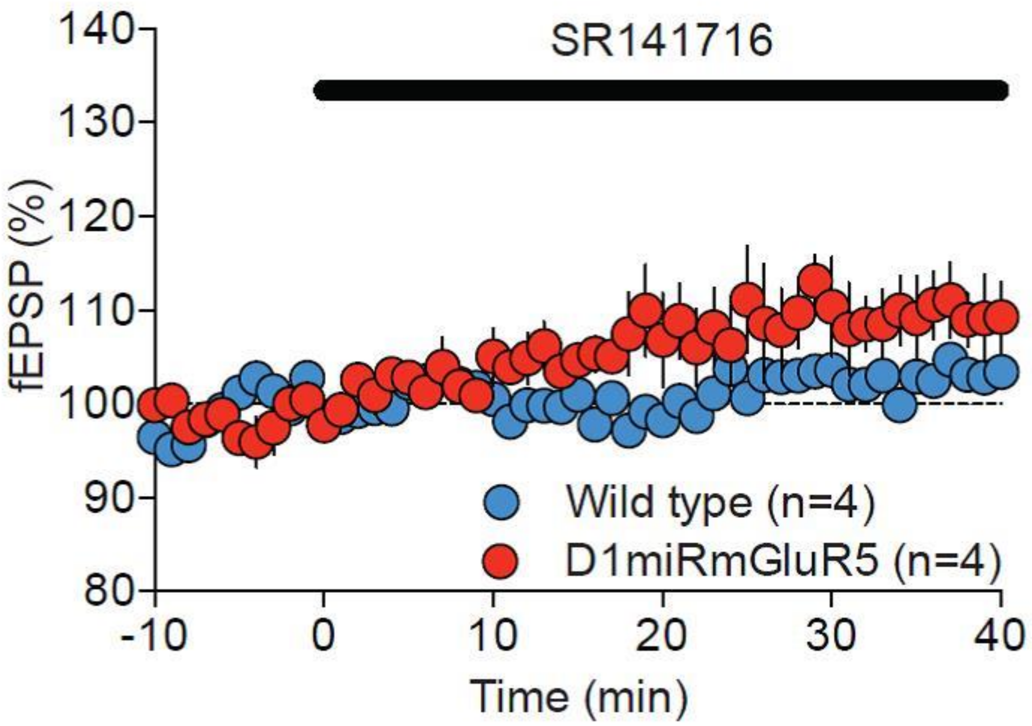

Figure S5. Tonic CB1R activation is minimal in both wild-type and D1^{miR}mGluR5 mice, related to Figure 3.

The cannabinoid inverse agonist SR141716A had no effect on baseline transmission in wild-type (blue) or D1^{miR}mGluR5 (red) mice. Average time course of mean EPSCs is represented as percentage of the basal value. All data represent mean ± SEM. Two-way ANOVA, $*P < 0.05$ vs. D1^{miR}mGluR5 mice.

Supplemental Figure 6

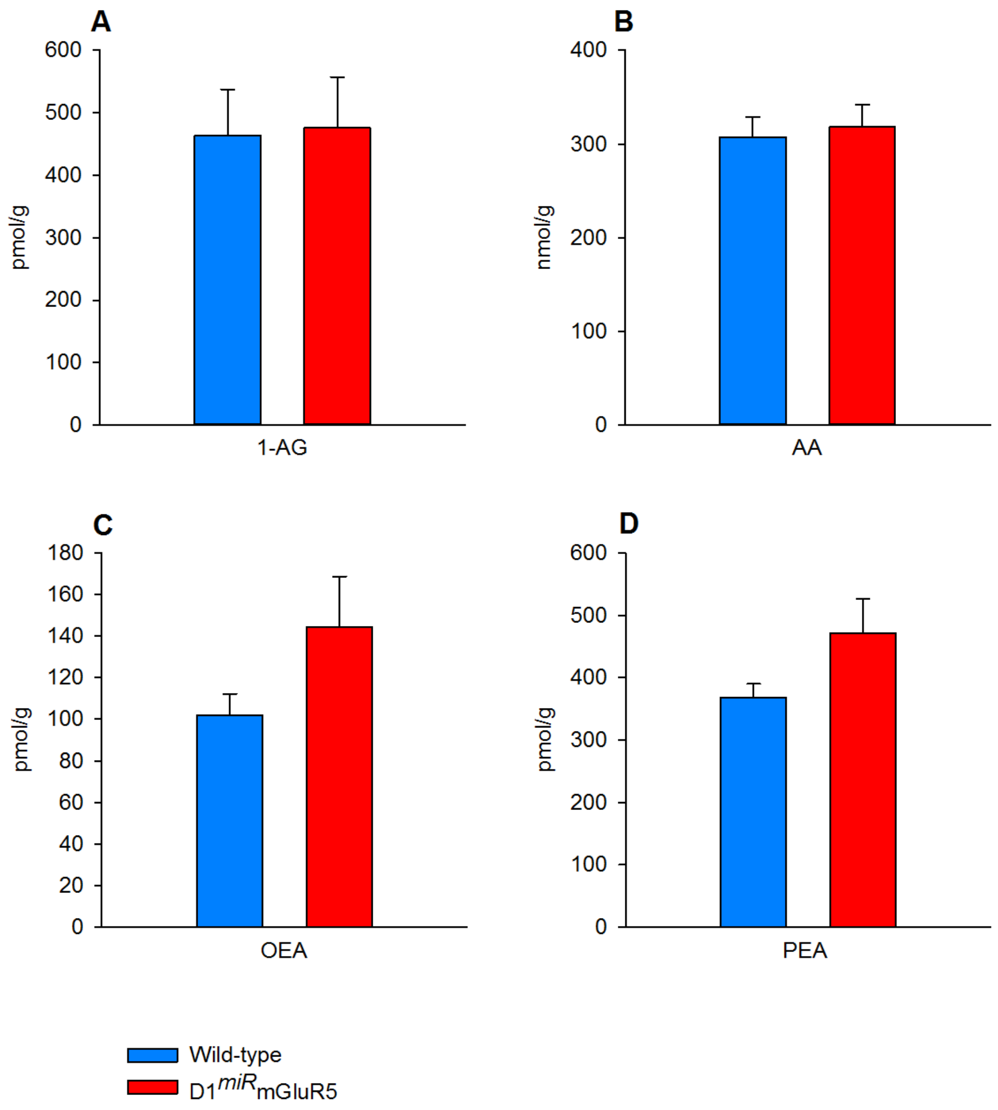

Figure S6. Basal eCB and congeners concentrations in the NAc of wild-type and D1^{miR}mGluR5 mice, related to Figure 3.

1-arachidonoyl glycerol (1-AG, (A)), arachidonic acid (AA, (B)), oleoylethanolamide (OEA, (C)) and palmitoylethanolamide (PEA, (D)) levels in NAc of wild-type (n=10) and D1^{miR}mGluR5 mice (n=10). Under basal, non-stimulated conditions, eCB and congeners levels are similar in both genotypes (Student's *t*-test, 1-AG: $t_{(18)}$=-0.1, *P* =0.9; AA: $t_{(18)}$= -0.3, *P* =0.7; OEA: $t_{(18)}$=-1.7, *P* =0.1; PEA: $t_{(18)}$= -1.8, *P* =0.1). Data represent picomoles or nanomoles/gram wet tissue ± SEM.

Supplemental Figure 7

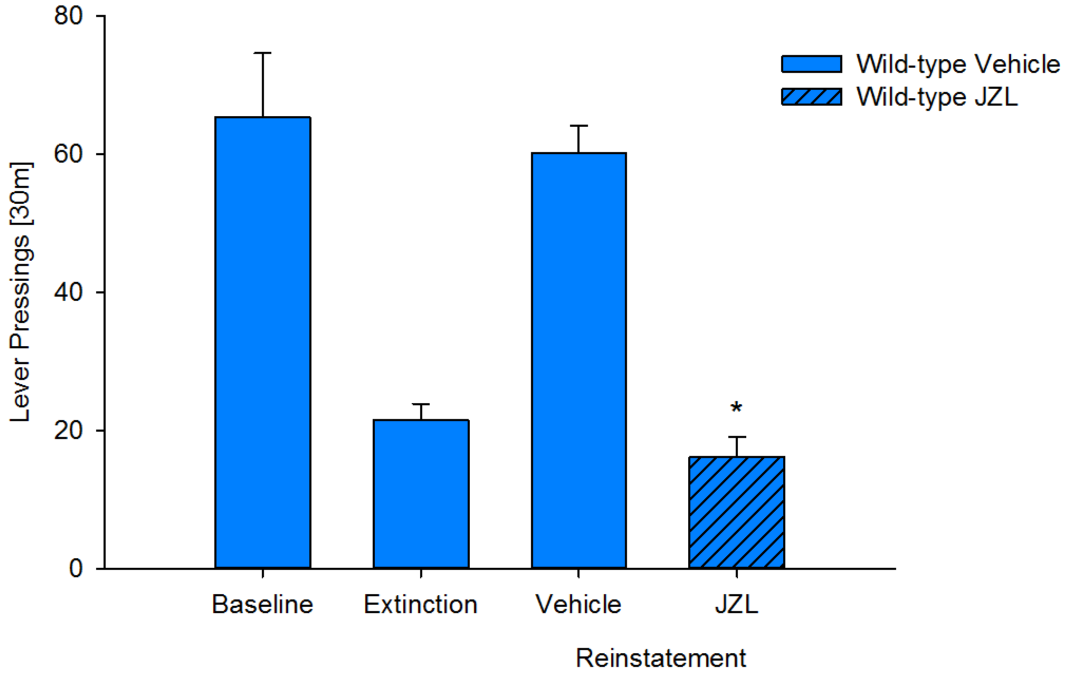

Figure S7. Effect of JZL administration on saccharin seeking behavior in wild-type mice, related to Figure 4.

In wild type mice (n=14), administration of JZL (16 mg/kg, i.p.) attenuated cue-induced reinstatement of saccharin-seeking behavior ($t_{(13)}$=9.2, P<0.0001). All data represent mean ± SEM. Student's *t*-test, **P* < 0.001 vs. vehicle treatment.

Transparent Methods

Procedures for this study complied with the regulations covering animal experimentation within the European Union (European Communities Council Directive 86/609/EEC) and Germany (Deutsches Tierschutzgesetz) and the experiment was approved by the German animal welfare authorities (Regierungspräsidium Karlsruhe).

Animals

D1^{miR}mGluR5 and wild-type male mice (6-8 weeks at the beginning of the experiments) were generated, genotyped and bred at the Central Institute of Mental Health in Mannheim. Short hairpin RNAs were designed using the sFold (sTarMir) and BLOCK-IT RNAi Designer (Invitrogen) software packages and tested in cell culture for knock-down (KD) efficiency of mGluR5 mRNA. BLOCK-iT Pol II miR RNAi Expression vector kit with GW/EmGFP-miR vector (Invitrogen) was used to insert synthetic oligos to artificial miRNA context. The construct was recombined into a bacterial artificial chromosome (BAC; RP24–179E13; Children's Hospital Oakland Research Institute, Oakland, CA) harboring the mouse D1R gene. The BAC was purified, the vector sequences were removed, and the transgene was injected into the pronuclei of fertilized oocytes from C57BL/6N mice. Experimental animals were generated by backcrossing of D1^{miR}mGluR5 transgenic mice to C57BL/6N line. Transgenic animals were genotyped using the following primers: ACGTAAACGGCCACAAGTTC, AAGTCGTGCTGCTTCATGTG (Novak et al., 2010). All animals were singly housed in standard hanging cages at 21 ± 1°C and 50 ± 5% relative humidity on a 12 h light/dark cycle, with lights on at 7:00 A.M. Animals were provided with standard rodent food and tap water *ad libitum*. Animals were handled on a daily basis before starting the experiments. Experiments were conducted in accordance with European Union guidelines on the care and use of laboratory animals.

Behavioral experiments

D1^{miR}mGluR5 and wild-type mice were tested for basic phenotype, cue-induced reinstatement of ethanol, saccharin or sucrose operant self-administration, and intracranial self-stimulation (ICSS). All experiments were performed during the active, dark phase of the day, between 9:00 and 14:00 h.

Basic Phenotype.

Characterization of locomotor, anxiety and short-term memory. The tests performed were home cage activity, habituation to the activity box, elevated plus maze paradigm, light-dark box, novelty-induced grooming and the free-choice exploration paradigm in Y-maze. All tests were performed by trained observers blind for genotypes. Devices used for all behavioral studies were carefully cleaned with a diluted acetic acid solution between animals to prevent olfactory cues.

Home cage activity. Locomotor activity in the home cage was monitored by connecting an infrared sensor (Mouse-E-Motion; Infra-E-Motion GmbH, Henstedt-Ulzburg, Germany). A Mouse-E-Motion device was placed above each cage (30 cm from the bottom), so that the mouse could be detected at any position inside the cage. The device was sampling every 4 s whether the mouse moved or not. The sensor could detect body movements of the mouse of ≥1.5 cm from one sample point to the next. Monitoring of locomotor activity started before the beginning of the experiments and lasted for 4–5 days, and data were collected every 4 h to measure the circadian pattern of motor activation.

Habituation to the activity box. This test was used to assess animal exploratory activity and reactivity to novel environment and to evaluate the effects of habituation mechanisms. Animals were placed in activity chambers in which locomotor activity was measured every min for a period of 30 min under novelty and familiarity (4 consecutive more days) conditions. Clear Plexiglas boxes of 40 cm in diameter and 40 cm in height were used, and the

locomotor activity was measured with a TruScan activity monitoring system (Coulbourn Instruments, Allentown, PA, USA).

Elevated plus maze. The plus maze consisted of 2 open arms and 2 enclosed arms extending from a central platform. The maze was elevated 50 cm above and illuminated from the top at 60 lux. Each mouse was placed at the intersection of the 4 arms of the maze and allowed to explore all 4 arms freely for 5 min, and the behavior was recorded and measured by the Noldus/EthoVision 3.1 monitoring system Wageningen, The Netherlands).

Light-dark box. The light-dark box test consisted of black and white plexiglass (45×20×27 cm) box. The dark compartment (15×27 cm) was cover and the light compartment (30×27 cm) remained open, and was kept at a luminosity of 350 lux. A door was located in the wall between the two chambers allowing free access between the light and dark compartments. Each mouse was placed in the dark chamber and was allowed to explore the box for 5 minutes, and the behavior was recorded and measured by the Noldus/EthoVision 3.1 monitoring system (Wageningen, The Netherlands).

Novelty-induced grooming. We selected novelty-induced grooming, as it represents a characteristic behavior associated with selective stimulation of D1 receptors. Mice were handled and placed in a glass observation box of 30x40x30 cm. and the time spent grooming and the number of oral stereotypies was video tracked over 10 min.

Free-choice exploration paradigm in Y-maze. This test studies working and short-term memory based in a simple measurement of novelty recognition. The test is based on the rodent’s innate curiosity to explore novel areas, and it is not biased by incentives/reinforcers such as food, etc. This test was used to assess preference and/or habituation to novelty and spatial memory. The apparatus consisted of three arms of black plastic forming a "Y." Mice were placed into one of the arms of the maze (start arm) and allowed to explore only another arm of the maze for 5 min. Two min and 30 min after the first exploration interval, mice were returned to the start arm and allowed to explore freely all three arms of the Y-maze for 5 min.

The number of entries into and the time spent in each arm was recorded and measured by the Noldus/EthoVision 3.1 monitoring system (Wageningen, The Netherlands).

Operant self-administration, extinction and cue-induced reinstatement of ethanol and saccharin seeking.

Mice were trained and tested in eight operant chambers (TSE Systems, Bad Homburg, Germany), operated with operant behavior system (TSE Systems). Each chamber had two ultrasensitive levers (required force, ≤1 g) on opposite sides: one functioning as the actuve and one as the inactive lever. Next to each lever, a front panel containing the visual stimulus was installed above a drinking microreservoir. When the programmed ratio requirements were met on the active lever, 10 µl of the solution were delivered into a microreservoir, and the visual stimulus was presented via a light located on the front panel. Responses on the inactive lever were recorded but had no programmed consequences. A microcomputer controlled the delivery of fluids, presentation of auditory and visual stimuli, and recording of the behavioral data.

Conditioning phase. Mice were trained to self-administer 10% ethanol (v/v), 0.2% saccharin (w/v) or 3% sucrose (w/v) in 30 min daily sessions on a fixed ratio 1 schedule of reinforcement, where each response resulted in delivery of 10 µl of fluid. A contextual stimulus predicting reward availability was presented during the self-administration sessions. The contextual stimulus consisted of a gray, smooth floor (S^+). In addition, each lever press resulting in delivery of fluid was paired with the illumination of the chamber's cue light for 5 s (CS^+). Concurrently with the presentation of these stimuli, a 5 s time-out period was in effect, during which responses were recorded but not reinforced. Criteria for the conditioning were met at stable baseline lever pressing for 3 consecutive days, with no significant differences in lever pressing.

Extinction phase. After the last conditioning day, mice were subjected to 30-min extinction sessions. Responses at the lever activated the delivery mechanism but did not result in the

delivery of liquids or the presentation of the response-contingent cue (light). The criteria for extinction were established at 40% of the baseline lever responses for 3 consecutive days.

Reinstatement testing. Reinstatement tests began the day after the last extinction session and lasted 30 min. In ethanol, saccharin or sucrose-trained mice, cue-induced reinstatement was tested under conditions identical to those during the conditioning phase, except that the fluid was not made available.

Intracranial self-stimulation.

Mice were anesthetized with 1.5-1.8% of isoflurane (CP-Pharma, Burgdorf, Germany), stereotaxically implanted with insulated monopolar stainless steel electrodes (28 mm diameter) (Plastics One, USA) to the right medial forebrain bundle in the lateral hypothalamus (coordinates from Bregma: anterior (AP) -1.2, lateral (ML) +1, ventral (DV) −5.4), and trained to respond for brain stimulation reward (BSR).

During each testing session, mice responded during three consecutive series of 15 descending frequencies (.05 log10 steps). Maximum control rate (MCR), and total number of stimulations were calculated from the average of the second and third series. Stimulation seeking and extinction components were calculated from the total number of stimulations during the first 5 highest frequencies and the remaining 10, respectively.

Surgery and intra-NAc microinfusions.

Mice were anesthetized with 1.5-1.8% of isoflurane (CP-Pharma, Burgdorf, Germany). Unilateral cannulae were implanted under stereotaxic guidance (David Kopf Instruments, Tujunga, USA) aimed at the nucleus accumbens core (from Bregma: anterior (AP) +1.65, lateral (ML) ±0.9, ventral (DV) −4.2). Stainless steel cannulae (-4.2 mm, 26 gauge) were used. Cannulae were secured with cement (Super-Bond C&B, Sun Medical, Moriyama, Japan), and a 33-gauge stainless steel stylets were inserted into the length of each guide cannula prevent blockade and contamination. Cannulae were implanted at the end of the self-

administration training phase, and mice had 3-4 days of recovery. For microinfusions, mice were gently restrained, stylets were removed and injectors (33-gauge stainless steel tubing) were lowered 0.1mm beyond the tip of the guide cannula into the accumbens core and were attached via polyethylene tubing (PE20) to 10 µl Hamilton syringes. Infusions of 0.5 µl were delivered by a syringe pump (Harvard Apparatus, Holliston, USA) and were given over 2min (flow rate 0.25µl/min) to limit injection spread into neighboring brain areas, as well as to minimize diffusion up the injector track. To ensure complete diffusion, injectors were removed 1min after completion of the infusion.

Drugs

For the behavioral experiments, ethanol dilution (10% w/v) was made up with 95% ethyl alcohol and water. Sodium saccharin (Sigma Chemical Co., Germany) or sucrose were added to water to achieve 0.2% and 3% (w/v), respectively. Systemic, intraperitoneal treatments included AM251 (0.3 mg/kg), MTEP (20 mg/kg), JZL184 (16 mg/kg, Sigma Chemical Co., Germany) and CP55, 940 (20 µg/kg) suspended with 2–3 drops of Tween 80 in saline as vehicle and cocaine (20 mg/kg) dissolved in saline. AM251 (1µg/0.5µl), JZL (1.6 and 3µg/0.5µl) were dissolved in water administered in the NAc core.

Drugs were administered 40min (MTEP, AM251 and JZL184, i.p.), 24h (cocaine and CP55, 940, i.p.) or 1h (AM251 and JZL184, intra-accumbal) before the reinstatement, self-administration tests or in the homecage. For the operant tests, drug administrations were conducted every third day using a counterbalanced design.

Statistical analyses

Statistical analyses were performed by ANOVA with Newman-Keuls test for post-hoc comparisons, Mann Whitney U-test or Student’s *t*-test. Significance was set at $P<0.05$.

Electrophysiological Experiments

Slice preparation

Nucleus accumbens (NAc) slices were prepared as follows. Briefly, mice were anesthetized with isoflurane and decapitated. The brain was sliced (300 μm) in the coronal plane (Integraslice, Campden Instruments, Leicester, U.K.) and maintained in artificial cerebrospinal fluid (ACSF) containing 126 mM NaCl, 2.5 mM KCl, 2.4 mM $MgCl_2$, 1.2 mM $CaCl_2$, 18 mM $NaHCO_3$, 1.2 mM NaH_2PO_4 and 11 mM glucose, equilibrated with 95% O_2/5% CO_2 at 4°C. Slices were then stored for 30 min at 32–35°C and at 22 ± 2 °C until recording in ACSF.

Electrophysiology

Whole-cell patch-clamp and extracellular field recordings were made from medium spiny neurons respectively, in coronal slices of mouse NAc (Deroche et al., 2020). For recording, slices were superfused (2ml/min) with ACSF. All experiments were done at 32–35 °C. The ACSF contained picrotoxin (100 μM) to block GABA-A receptors. To evoke synaptic currents, 150-200 μs stimuli were delivered at 0.1Hz through an ACSF-filled glass electrode placed at a distance > 150 µm in the dorsomedial direction (ventral striatum recordings). For extracellular field experiments, the recording pipette was filled with ACSF. The glutamatergic nature of the field excitatory postsynaptic potential (fEPSP) was confirmed at the end of each experiments using the ionotropic glutamate receptor antagonist 6, 7-dinitroquinoxaline-2,3-dione (DNQX, 20 μM), that specifically blocked the synaptic component without altering the non-synaptic component (data not shown). LTD was induced by low frequency stimulation of 10 minutes at 10 Hz. For whole-cell patch-clamp, pyramidal neurons in PFC layer V/VI and medium spiny neurons of NAc were visualized using an infrared microscope (BX-50WI or BX-51WI, Olympus). Experiments were made with electrodes containing 128mM potassium

gluconate (KGlu), 20mM NaCl, 1mM $MgCl_2$, 1mM EGTA, 0.3mM $CaCl_2$, 2mM Na^{2+}-ATP, 0.3mM Na^{+}-GTP, 10mM glucose buffered with 10mM HEPES, pH 7.3, osmolarity 290mOsm. Electrode resistance was 4–6MOhm. If access resistance (no compensation, <25MOhm) changed by >20%, the experiment was rejected. To perform the voltage-clamp experiments, evoked EPSCs were recorded at -70mV.

Retrograde tracing

Under general ketamine-xylazine anesthesia and stereotactic control, 80 nl of red or green fluorescent latex microspheres (Lumafluor, Naples, F) were pressure injected bilaterally in the ventral mesencephalon (bregma: -3.3mm, lateral: 0.6mm, ventral:4.8mm) or Ventral pallidum (bregma:0.15mm, lateral:1.8mm ventral:4.8mm). Animals were sacrificed 14-28 days after injections and brain slices prepared for electrophysiology.

Retrogradely labeled direct and indirect pathway MSNs were visualized by infrared-differential interference contrast (IR-DIC) and epifluorescence microscopy (Olympus BX-51WI).

Data acquisition and analysis

The potential reference of the amplifier was adjusted to zero before breaking into the cell or entering the slice. Data were recorded on a MultiClamp 700B or Axopatch 200B (Axon Instruments), filtered at 2kHz, digitized (20kHz, DigiData 1440A or 1322A, Axon Instrument), collected using Clampex 10.2 and analyzed using Clampfit 10.2 (all from Molecular Device, Sunnyvale, USA). Analysis of both area and amplitude of fEPSP and EPSCs was performed (graphs depict amplitudes for patch clamp experiments and areas for field recordings). The magnitude of LTD was calculated 25-30 minutes after tetanic protocol as percentage of baseline responses.

The fitting of concentration response curves was calculated according to y={ymax2ymin/1+(x/EC50)n}+ymin (where ymax=response in the absence of agonist, ymin=response remaining in presence of maximal agonist concentration, x=concentration, EC50=concentration of agonist producing 50% of the maximal response and n=slope) with GraphPad Prism 5.0 (GraphPad Software Inc., La Jolla, CA).

Drugs

Drugs were added at the final concentration to the ACSF. Picrotoxin was from Sigma (St. Quentin Fallavier, France). DNQX was from the National Institute of Mental Health's Chemical Synthesis and Drug Supply Program (Rockville, MD, USA). LY379268, JZL184 and CP55, 940 were from Tocris (Bristol, UK).

Statistical analysis

The value *n* corresponds to the number of animals. All values are given as mean ± SEM. and statistical significance was set at $P < 0.05$. Statistical analysis (ANOVA or Mann Whitney U-test), was performed with GraphPad Prism 5.0 (GraphPad Software Inc., La Jolla, CA).

Immunohistochemistry for electron microscopy

Preembedding immunocytochemical method for electron microscopy. Animals were deeply anesthetized by i.p. injection of a mixture of Nembutal (5mg/100g body weight; Abbott Laboratories Inc., IL, USA) and urethane (130mg/100g body weight; Sigma-Aldrich, St. Louis, MO, USA). They were transcardially perfused with PBS (0.1 M, pH 7.4) and then fixed with 250 ml of 4% formaldehyde, 0.1% glutaraldehyde and 0.2% saturated picric acid in PB (0.1M, pH 7.4). Perfusates were used at 4°C. Tissue blocks were extensively rinsed in 0.1 M PBS (pH 7.4). Coronal brain vibrosections were cut at 50 µm and collected in 0.1 M PBS (pH 7.4) at RT. Sections containing the nucleus accumbens were preincubated in a blocking

solution of 10% bovine serum albumin (BSA), 0.1% sodium azide and 0.02% saponin prepared in Tris-HCl buffered saline (TBS, pH 7.4) for 30 minutes at RT. A preembedding silver-intensified immunogold method was used for the localization of CB1R, DAGL-α and MAGL proteins. The primary polyclonal antibodies used in this study were: polyclonal goat antibody to CB1R (2μg/ml; CB1R-Go-Af450-1; Frontier Science Co. Ltd; 1-777-12, Shinko-nishi, Ishikari, Hokkaido, Japan), polyclonal goat antibody to DAGL-α (2μg/ml; DAGL-α-Go-Af1080-1; Frontier Science Co. Ltd; 1-777-12, Shinko-nishi, Ishikari, Hokkaido, Japan) and polyclonal rabbit antibody to MAGL (1:100; Cayman Chemical Company, Michigan 48108, USA). Accumbens sections were incubated with the primary antibodies in 10% BSA/TBS containing 0.1% sodium azide and 0.004% saponin on a shaker for 1 day at RT. After several washes in 1% BSA/TBS, tissue sections were incubated in the secondary 1.4 nm gold-labeled rabbit anti-goat IgG and goat anti-rabbit IgG (Fab' fragment, 1:100, Nanoprobes Inc., Yaphank, NY, USA), depending on the primary antibodies, in 1% BSA/TBS with 0.004% saponin on a shaker for 4 hours at RT. Thereafter, the tissue was washed in 1% BSA/TBS overnight at 4ºC and postfixed in 1% glutaraldehyde in TBS for 10 minutes at RT. Following washes in double-distilled water, gold particles were silver-intensified with a HQ Silver kit (Nanoprobes Inc., Yaphank, NY, USA) for about 12 minutes in the dark and then washed in 0.1X PBS (pH 7.4). Stained sections were osmicated (1% OsO4 in 0.1X PBS, pH 7.4, 20 minutes), dehydrated in graded alcohols to propylene oxide and plastic-embedded flat in Epon 812. 80nm ultrathin sections were collected on mesh nickel grids, stained with uranyl acetate and lead citrate, and examined in a Philips EM2008S electron microscope. Tissue preparations were photographed by using a digital camera coupled to the electron microscope. Figure compositions were scanned at 500 dots per inch (dpi). Labeling and minor adjustments in contrast and brightness were made using Adobe Photoshop (CS, Adobe Systems, San Jose, CA, USA).

Analysis of the proportion of immunolabeled profiles in nucleus accumbens. Sections with nucleus accumbens processed for the localization of CB1R, DAGL-α and MAGL with preembedding immunocytochemistry were used for semi quantitative analysis. Tissue showing good and reproducible silver-intensified gold particles were cut at 80 nm. Electron micrographs (10,000-25,000X) were taken from grids (132 μm side). To avoid false negatives, only ultrathin sections in the first 1.5 µm from the surface of the tissue block were examined. Positive labelling was considered if at least one immunoparticle was over postsynaptic or presynaptic membranes, within approximately 30 nm from the membranes, or within the cellular profile in case of MAGL. Percentages of immunopositive profiles were analyzed and displayed using a statistical software package (GraphPad Prism 4, GraphPad Software Inc, San Diego, USA).

Measurement of endocannabinoid levels by LC-MS/MS analysis

Standards for endocannabinoid measurements (anandamide (AEA), 2-arachidonoyl glycerol (2-AG), 1-arachidonoyl glycerol (1-AG), oleoylethanolamide (OEA), palmitoylethanolamide (PEA), arachidonic acid (AA), and their deuterated analogues AEA-d_4, 2-AG-d_5, 1-AG-d_5, OEA-d_2, PEA-d_4, and AA-d_8) were obtained from Cayman Chemicals (Ann Arbor, Michigan, USA). Water (H_2O), acetonitrile (ACN), formic acid (FA), ethylacetate and hexane (all of Fluka LC-MS grade) were obtained from Sigma-Aldrich (Munich, Germany) and Carl Roth (Karlsruhe, Germany). All stock solutions, intermediate dilutions and calibration standards were made up with ACN at appropriate concentration levels. For the measurement of endocannabinoid levels, punches were kept frozen at -80°C. Samples were weighed in the extraction tubes, spiked with acetonitrile (ACN) containing the internal standards, homogenized in ice-cold 0,1 M formic acid using the TissueLyser II (Qiagen, Hilden, Germany) and extracted with ethylacetate/hexane (9:1, v/v). The tubes were vortexed for 30 seconds, and centrifuged for 10 min at 10000 g and 4°C. The upper (organic) phase was

removed, evaporated to dryness under a gentle stream of nitrogen at 37°C and re-dissolved in ACN/H_2O (1:1, v/v). The LC-MS/MS analysis was then performed on a LC-MS/MS system consisting of a 5500 QTrap triple-quadrupole linear ion trap mass spectrometer equipped with a Turbo V Ion Source (AB SCIEX, Darmstadt, Germany) with a “positive-negative switching” mode, an Agilent 1200 series LC system (degasser, pump, and thermostated column compartment; Agilent, Waldbronn, Germany), and a CTC HTC PAL autosampler (CTC Analytics AG, Zwingen, Switzerland). ECBs and related compounds were separated on a Phenomenex Luna 2.5 µm C18(2)-HST column combined with a SecurityGuard pre-column (Phenomenex, Aschaffenburg, Germany) with solvents A: 0.1% FA in 20:80 ACN/water (v/v), and B: 0.1% FA in ACN, using the following gradient: linear from 55-90% B (0-2 min), then held at 90% B (2-7.5 min), and re-equilibrated at 55% B (7.5-10 min). The column temperature was 25°C, the LC flow rate 0.3 ml/min, and the injection volume 20 µL. Positive and negative ions were analyzed simultaneously by combining two experiments in 'positive-negative-switching' mode. The Turbo V Ion Source was operated with the electrospray ('TurboIon') probe with nitrogen as curtain and nebulizer gas, and using the following settings: Temperature 550°C, curtain gas 40 psi, GS1 50 psi, GS2 50 psi, capillary voltage -4500 V (negative) and +5500 V (positive). The following precursor-to-product ion transitions were used for multiple-reaction monitoring (MRM): positive: AEA m/z 348.3 -> 62.1, AEA-d_4 m/z 352.3 -> 66.1, 2-AG/1-AG m/z 379.1 -> 287.2, 2-AG-d_5/1-AG-d_5 m/z 384.2 -> 287.2, OEA m/z 326.2 -> 62.1, OEA-d_2 m/z 328.2 -> 62.1, PEA m/z 300.2 -> 62.1, PEA-d_4 m/z 304.2 -> 62.1; negative: AA m/z 303.1 -> 259.1, AA-d_8 311.0 -> 267.0. Data acquisition and analysis were performed using Analyst software (version 1.5.1; AB SCIEX).

Supplemental References

Deroche, M.A., Lassalle, O., Castell, L., Valjent, E., Manzoni, O.J. (2020). Cell-type and endocannabinoid specific synapse connectivity in the adult nucleus accumbens core. J Neurosci. 40(5), 1028-1041. Epub 2019 Dec 12.

Novak, M., Halbout, B., O'Connor, E.C., Rodriguez Parkitna, J., Su, T., Chai, M., Crombag, H.S., Bilbao, A., Spanagel, R., Stephens, D.N., Schütz, G., and Engblom, D. (2010). Incentive learning underlying cocaine-seeking requires mGluR5 receptors located on dopamine D1 receptor-expressing neurons. J. Neurosci. 30, 11973-11982.
